# Symbiotic Activity of Pea (*Pisum sativum*) after Application of Nod Factors under Field Conditions

**DOI:** 10.3390/ijms15057344

**Published:** 2014-04-29

**Authors:** Anna Siczek, Jerzy Lipiec, Jerzy Wielbo, Dominika Kidaj, Paweł Szarlip

**Affiliations:** 1 Institute of Agrophysics, Polish Academy of Sciences, P.O. Box 201, 20-290 Lublin, Poland; E-Mails: asiczek@ipan.lublin.pl (A.S.); pszarlip@ipan.lublin.pl (P.S.); 2 Department of Genetics and Microbiology, Maria Curie-Skłodowska University, Akademicka 19 st, 20-033 Lublin, Poland; E-Mails: jerzy.wielbo@poczta.umcs.lublin.pl (J.W.); donia81@gmail.com (D.K.)

**Keywords:** nodules, nitrogenase activity, seed yield, protein yield, pea shoots, weather conditions

## Abstract

Growth and symbiotic activity of legumes are mediated by Nod factors (LCO, lipo-chitooligosaccharides). To assess the effects of application of Nod factors on symbiotic activity and yield of pea, a two-year field experiment was conducted on a Haplic Luvisol developed from loess. Nod factors were isolated from *Rhizobium leguminosarum* bv. *viciae* strain GR09. Pea seeds were treated with the Nod factors (10^−11^ M) or water (control) before planting. Symbiotic activity was evaluated by measurements of nitrogenase activity (acetylene reduction assay), nodule number and mass, and top growth by shoot mass, leaf area, and seed and protein yield. Nod factors generally improved pea yield and nitrogenase activity in the relatively dry growing season 2012, but not in the wet growing season in 2013 due to different weather conditions.

## 1. Introduction

In a nitrogen-limiting environment, legumes can establish nitrogen-fixing symbiosis with bacteria from the family Rhizobiaceae. In the early stage of symbiosis, recognition between plant and bacterial partners involves exchange of molecular signals. Flavonoids secreted by host plant roots with the NodD protein induce the biosynthesis of specific bacterial lipochitin oligosaccharides called nodulation factors (Nod factors, LCO, lipo-chitooligosaccharides). A single rhizobial strain produces populations of these metabolites that consist of two to approximately 60 different individuals [1]. Qualitative and quantitative aspects of Nod factor populations are strain-specific. These molecules induced nodulation processes at the host plant epidermis, cortex and pericycle, such as deformation of root hairs, membrane potential depolarization, and formation of nodule primordia [1]. Application of Nod factors caused transient increases in the cytosolic calcium concentration in root hairs and improved calcium uptake into soybean leaves [2], rapid changes in the pattern of microtubules in root hairs of *Medicago sativa* [3], and a localized and temporary inhibition of auxin transport, which subsequently leads to accumulation of auxin at the site of nodule formation [4].

Several studies investigated the influence of Nod factors that were applied on seeds or into growth medium under controlled growth chamber conditions. In the study by Souleimanov *et al.* [5], addition of Nod factor to the hydroponic medium had a positive effect on soybean growth by increasing root length and surface area. More recently, Kidaj *et al.* [6] observed in a pot experiment that Nod application on pea and vetch seeds significantly enhanced the percentage of germinated seeds, root size, nodule number, and symbiotic performance whereas the total nitrogen content in the plants remained unchanged. An increased nodule number and shoot growth following Nod application on seeds was also observed in clover grown in a pot soil [7]. Foliar application of Nod factors increased the photosynthesis rate up to 10%–20% over the control and this was connected with an increase in stomatal conductance of soybean [8–10]. Prithiviraj *et al.* [11] demonstrated that the Nod factor of *Bradyrhizobium japonicum* enhanced seedling emergence of plants belonging to diverse botanical families (corn, soybean, cotton) under field conditions. In a greenhouse experiment, they observed that Nod factors considerably improved emergence and early growth (leaf area, root length, shoot and root weight) of corn 15 days after planting. Recent studies in a growth-chamber environment showed that treatment of pea seeds with Nod factors before planting resulted in a significant increase in nitrogenase activity and total plant nitrogen content [12].

Literature review showed that the effect of Nod factors on plants was mainly focused on early growth parameters and the investigations were conducted mostly under laboratory, controlled conditions. However, the effect of Nod factors on symbiotic activity under natural, field conditions remains largely unknown. Thus, the objective of this study was to evaluate nitrogenase activity, nodulation and pea yield in a field experiment after seed treatment with Nod factors.

## 2. Results and Discussion

### 2.1. Nodulation Characteristics

Compared to the control, treatment of pea seeds with the Nod factors significantly affected nodulation parameters (Table 1). Application of the Nod factors doubled the nodule number in both years and nodule mass by 67% (*p* < 0.05) and 23% in 2012 and 2013, respectively. However, there was no significant effect of the Nod factors on the mass of one nodule. These results confirmed the beneficial effect of application of the Nod factors on nodule parameters observed in earlier studies under controlled growth conditions [5,6].

The effect of the Nod factors on nitrogenase activity depended on the study year. The Nod factors increased (*p* < 0.05) nitrogenase activity in comparison with the control treatment in 2012 (nearly 1.5 times), but in 2013 the Nod effect was not significant. However, nitrogenase per g^−1^ of nodule dry weight was significantly greater in the Nod treatment than in the control in both years. There was no significant effect of the Nod factors on nitrogenase activity per nodule. All nitrogenase indicators were significantly affected by the study year (Table 3) and the effects of Nod and Y × Nod interaction were significant for nitrogenase per 4.5 dm^−3^ of soil and 1 g of nodule dry weight. The beneficial effect of the Nod factors on nitrogenase activity in the relatively dry growing season 2012 (Table 5) was greater than in an earlier pot experiment with pea under near optimum water supply [12]. The increasing nodule activity in response to the Nod factors could be partially explained by symbiotic competitiveness of the bacterial strains [6].

The different weather conditions during the pea growing season in 2012 and 2013 influenced appreciably nodulation parameters. The relatively dry period during April–June in 2012 resulted in fewer but larger nodules than in 2013. In a study by King and Purcell [13], larger nodules under water deficit had a higher content of photosynthates and water, and exhibited greater oxygen permeability than smaller nodules due to greater amounts of nitrogen fixing tissue. Moreover, the number of nodules can be controlled by plants via a system called autoregulation of nodulation (AON) [14], which is activated by both rhizobia and Nod factors [15].

The results in Table 1 indicate that inoculation with the Nod factors led to an increased nodule number from 2012 to 2013 but this increase was not reflected in greater nitrogenase activity. This can be partly due to a preferential uptake of soil nitrogen under wet conditions in 2013, which increased nitrogen accessibility for plants [16,17]. In general, the preferential use of soil nitrogen *vs.* fixed nitrogen by plants results from lower energy requirement. Besides, before plant sampling for nitrogenase determination, we observed standing water locally in soil depressions that may have reduced nitrogenase activity as an effect of oxygen deficit [18].

### 2.2. Shoot Parameters and Yield Components

Application of the Nod factors significantly increased shoot mass in 2012 and 2013 (Table 2) and leaf area in 2012 in pea at the flowering stage. The Nod factors increased the protein yield by 21% in the relatively dry growing season 2012 (*p* < 0.05). However, there were no significant differences between the treatments in the wet growing season 2013 for both the seed and protein yield. The year and Nod factors affected significantly both yield parameters (Table 3), and there were no Y × Nod interactions.

The beneficial effect of the Nod factors on pea productivity could be attributed to stimulation of cell division due to induction cell cycle genes and morphogenic activity [9,11,19] as well as promotion of seed germination and early growth of plants [5–7,20]. Some studies reported that Nod factors increased α-amylase activity in the seeds of Nod-treated corn [11] and changed balance in phytohormones (auxin and cytokinin) [4]. The promoting effect of Nod factors on growth and yield of leguminous plants might be further due to enhancing colonization of their roots by AM fungi [21] and their resistance to pathogens [22]. Irrespective of the Nod factors, the seed and protein yields were appreciably greater (by 53% and 54%, respectively) in 2013 than 2012 (Table 2), which can be ascribed to higher rainfalls during growing season.

Overall, the two-year study showed that the effect of Nod factors on the pea nodulation and productivity highly depended on weather conditions during growing season. The Nod factors increased nitrogenase activity and seed and protein yields of pea only in the dry-growing season. This implies that application of Nod factors in this experimental area can be advantageous for the pea productivity in growing seasons with less than average rainfalls.

## 3. Experimental Section

### 3.1. Study Site and Experimental Design

The two-year (2012–2013) field study was conducted in Lublin, Poland (51°15′N, 22°35′E). The soil, a Haplic Luvisol developed from loess, was under long-term (30 years) conventional tillage, with main tillage operations including pre-plough (10 cm depth) and harrowing, and moldboard plowing (20 cm depth). The characteristics of the experimental soil are presented in Table 4. The number of *Rhizobium leguminosarum* bv. *viciae* in the plough layer of the soil amounted to 1.8 × 10^3^ per gram of dry soil as determined using the most probable number technique modified by Martyniuk *et al.* [23], and was assessed as high and sufficient for nodulation [24]. This can be supported by the results of Drew and Ballard [25] indicating that nodulation occurred when the number of rhizobia ranged from barely 100 to 1.2 × 10^6^ per gram of soil, however, it was not significantly correlated with shoot dry weight. Accordingly, inoculation of pea seeds with symbiotic bacteria was omitted. The weather conditions differed during the two years (Table 5). The amount of rainfall during the pea growing season (April–July) was 168 mm in 2012 and 331 mm in 2013. The respective values were 31% lower and 36% higher than the long-term average (243 mm). The mean air temperatures during the pea growing season were similar in 2012 and 2013 (15.9 and 15.5 °C) and higher than the long-term average (13.7 °C).

Pea (*Pisum sativum* L. cv. Tarchalska) seeds were soaked for 30 min with the Nod factors (10^−11^ M) (Nod factors) or water (Control) before planting. The synthesis of rhizobial Nod factors by *R. leguminosarum* bv. *viciae* strain GR09 was induced by adding flavonoids. Flavonoids were extracted from sprouted pea seeds. Briefly, surface sterilized seeds were shaken in sterile water in darkness. Sprouted seeds were separated from water suspension and plant tissue debris was removed from the supernatant. Then, ethyl acetate was added to the supernatant and flavonoids were extracted from the aqueous phase. After evaporation of ethyl acetate, the pellet containing flavonoids was resolubilized in 95% ethanol and stored at 4 °C. R. *leguminosarum* bv. *viciae* cultures were grown overnight in liquid TY broth [26] to an OD_550_ of 0.6 and was then used to inoculate the liquid TY medium. The synthesis of the Nod factors was induced by adding pea seeds to the flavonoids at a final concentration of 10 μM, and then the rhizobial cultures were incubated with shaking. To isolate the Nod factors, the culture was centrifuged to remove bacterial cells, and the supernatant was extracted with *n*-butanol [11]. The organic fraction was separated and dried. A more detailed description of the procedure is provided in Siczek *et al.* [12].

To increase the concentration of the Nod factors in proximity of pea seedlings we inoculated the seeds, which resulted in a greater number of foci of cortical cell divisions and stimulated development of nodule primordia [27]. The plots (2 m × 3 m) were organized in four replicates, following the randomized complete block design. Fertilization was applied uniformly to all the plots at a rate of N-25, P-50, K-60, and Mg-35 kg·ha^−1^.

### 3.2. Plant Parameters and Nitrogenase Measurements

At the flowering stage, six pea plants from the control and Nod factor treatments were sampled and used for determination of shoot dry mass (after drying in 65 °C for 48 h) and leaf area (Image Analysis System, Delta-T Devices LTD, Cambridge, UK). Nitrogenase activity in root nodules was measured at the flowering stage by means of an acetylene reduction assay (C_2_H_2_). Root samples with attached nodules were taken from a row-crop, 0–15 cm soil layer in an area of 300 cm^2^ (in six replicates for both treatments) and washed. Afterwards, roots with nodules were immediately used for nitrogenase activity determination. The roots were placed in bottles and 10% (*v*/*v*) of gas from the bottles was replaced with acetylene. After 30 min incubation at room temperature, 1 mL of gas was sampled from the bottles and analyzed for ethylene concentration using a gas chromatograph Shimadzu GC14-B with FID (Flame Ionization Detector, Shimadzu, Kyoto, Japan). A 274.3 cm long stainless steel column was packed with carbosphere 80/100 mesh. The temperatures of the injector, column oven, and detector were 150, 230 and 230 °C, respectively. Helium was used as the carrier gas. The nitrogenase activity was calculated in C_2_H_4_ μmol·h^−1^ per 4.5 dm^3^ of soil. The amount of ethylene (μmol·h^−1^) was converted to individual nodules and 1 g of nodule dry weight (specific nitrogenase activity). The nodules were counted and dry mass (after drying in 65 °C for 48 h) was determined.

The seed protein content after harvest was determined on the basis of the nitrogen content assessed by means of the Kjeldahl method and was used to calculate protein yield.

### 3.3. Statistical Analysis

Statistical analysis was performed using STATISTICA 8.0 (StatSoft, Inc., Tulsa, OK, USA). Significant difference between mean values was determined by the *t*-test procedure. Statistical significance of the study year, Nod factors, and their interaction was assessed using a two-way ANOVA.

## 4. Conclusions

To our knowledge, this is the first study assessing the effects of Nod factors on symbiotic activity along with the yield of pea under field conditions. This study revealed a beneficial effect of application of the Nod factors on pea growth and nodulation, which was more substantial in the dry rather than wet growing season. Our results suggest possible application of Nod factors for increasing pea productivity and nitrogen fixation.

## Figures and Tables

**Table 1.** Nodulation parameters (4.5 dm^−3^ soil) in relation to Nod factors application in 2012 and 2013 (*n* = 6). Means followed by the same letter within the same parameter and the same year are not significantly different (*p* < 0.05).

Nodulation parameters	2012		2013	
	
	Control	Nod factors	Control	Nod factors
Nodules				

Number	30.3 b	63.4 a	83.5 b	166.8 a
Mass (mg)	122.4 b	204.5 a	319.3 a	395.4 a
Mass (mg·nodule^−1^)	3.70 a	2.98 a	3.14 a	2.27 a

Nitrogenase activity (C_2_H_4_ μmol·h^−1^)				

4.5 dm^−3^ soil	11.85 b	29.50 a	12.32 a	18.08 a
nodule^−1^	0.400 a	0.576 a	0.142 a	0.168 a
g^−1^ nodule dry weight	97.8 b	155.9 a	39.0 b	57.8 a

**Table 2.** Plant parameters in relation to Nod factors application in 2012 and 2013 (*n* = 6 for shoot and *n* = 4 for yield parameters). Means followed by the same letter within the same parameter and the same year are not significantly different (*p* < 0.05).

Plant parameters	2012		2013	
	
	Control	Nod factors	Control	Nod factors
Shoot mass (g·plant^−1^)	3.1 b	4.7 a	3.9 b	4.6 a
Leaf area (cm^2^·plant^−1^)	250 b	353 a	378 a	397 a
Seed yield (g·m^−2^)	416.4 b	482.5 a	671.0 a	705.8 a
Protein yield (g·m^−2^)	85.8 b	103.7 a	140.2 a	151.8 a

**Table 3.** *p* values for nodulation and plant parameters as related to year (Y), Nod factors (Nod) and their interactions.

Parameters	Y	Nod	Y × Nod
Nodules			

Number	<0.001	<0.001	0.023
Mass (mg)	<0.002	0.011	0.918
Mass (mg·nodule^−1^)	0.192	0.090	0.832

Nitrogenase activity (C_2_H_4_ μmol·h^−1^)			

4.5 dm^−3^ soil	0.007	<0.001	0.004
nodule^−1^	<0.001	0.069	0.167
g^−1^ nodule dry weight	<0.001	<0.001	0.027
Shoot mass (g·plant^−1^)	0.076	<0.001	0.028
Leaf area (cm^2^·plant^−1^)	<0.001	0.005	0.04
Seed yield (g·m^−2^)	<0.001	0.014	0.389
Protein yield (g·m^−2^)	<0.001	0.002	0.396

**Table 4.** Characteristics of the experimental soil (0–25 cm).

g·kg^−1^					mg·kg^−1^			pH H_2_O
	
Clay	Silt	Sand	C org	Total N *	P	K	Mg	
70	290	640	14.1	0.75	90	153	23	5.9

* as indicated by the Kjeldahl method.

**Table 5.** Weather conditions during 2012–2013.

Months	Rainfall (mm)			Air temperature (°C)		
	
	2012	2013	Long-term (50 years)	2012	2013	Long-term (50 years)
April	17	52	40.6	10.7	8.3	7.5
May	49	98	58.3	14.9	15.3	13.0
June	57	90	65.8	16.9	18.7	16.5
July	45	91	78.0	21.2	19.5	17.9
Annual	449	565	572	8.4	8.8	7.4
